# 3D X-ray computed tomography gray value and age model datasets of coral cores Baler 2 and 3 (Philippines)

**DOI:** 10.1016/j.dib.2021.106755

**Published:** 2021-01-14

**Authors:** Angel T. Bautista VII, Sophia Jobien M. Limlingan, Mary Margareth T. Bauyon, Arvin M. Jagonoy, Joseph Michael D. Racho, Jeff Darren G. Valdez, Araceli M. Monsada, Bee Jay T. Salon, Aldrin Jan E. Tabuso, John Kenneth C. Valerio, Edwin E. Dumalagan, Fernando P. Siringan

**Affiliations:** a Philippine Nuclear Research Institute – Department of Science and Technology (DOST-PNRI), Commonwealth Ave., Diliman, Quezon City 1101, Philippines; b Industrial Technology Development Institute – Advanced Device and Materials Testing Laboratory (DOST-ITDI ADMATEL), DOST Compound, General Santos Ave., Bicutan, Taguig City 1631, Philippines; c Marine Science Institute, University of The Philippines Diliman, Quezon City 1101, Philippines

**Keywords:** Coral core, 3D X-ray computed tomography, Sea surface temperature, Age dating, Philippines

## Abstract

The datasets here contain the 3D X-ray computed tomography (3DXCT) gray values and age models of coral cores Baler 2 and 3, taken from Baler, Aurora, Philippines. 3DXCT was used to analyze 5 mm-thick slabs of the coral cores. From the resulting 3DXCT images, gray values were determined per pixel from top to bottom of the slabs. The gray value profiles across the length of the slabs were then matched with records of sea surface temperature (SST) of the Baler site to construct the age model of the coral cores. Daily SST records from October 2018 to February 1982 were from the Optimum Interpolation Sea Surface Temperature or OISST [1,2], while monthly SST records from February 1982 to May 1945 were from the Extended Reconstructed Sea Surface Temperature or ERSST [3]. The gray value datasets of coral cores Baler 2 and 3 present historical records of the corals' response to changing environments through the years and may be used in studies related to such. An example of this can be seen in the relationship between coral gray values and SST. Furthermore, the age model datasets of Baler 2 and 3 serve as the basis for interpretation for all current and future studies on these coral cores. These datasets were originally produced for the research work titled “A historical record of the impact of nuclear activities based on ^129^I in coral cores in Baler, Philippines: an update” [4].

## Specifications Table

**Table:** 

Subject	Oceanography
Specific subject area	Paleoceanography, Corals
Type of data	Table Image Chart
How data were acquired	5 mm-thick coral slabs were analyzed using 3D X-ray Computed Tomography or 3DXCT (North Star Imaging X5000) in the Industrial Technology Development Institute – Advanced Device and Materials Testing Laboratory (ITDI-ADMATEL). Gray values were determined from top to bottom of the slabs. The gray value profile across the slabs' length was then matched with records of SST of the Baler site. Daily SST records from October 2018 to February 1982 were from the Optimum Interpolation Sea Surface Temperature or OISST [1,2], while monthly SST records from February 1982 to May 1945 were from the Extended Reconstructed Sea Surface Temperature or ERSST [3]. Matching of the gray values vs. SST was done through the QAnalySeries software [5]. QAnalySeries generated the age model for the Baler 2 and Baler 3 slabs after the matching step, based on the age dates of the SST records.
Data format	Raw Analyzed
Parameters for data collection	From the 3D images of the coral slabs, a 2D image slice was taken at 2.5 mm of the slab's thickness axis (i.e., at half-thickness). On the 2D image, gray values were then determined per pixel from top to bottom of the slabs.
Description of data collection	The general settings for the 3DXCT analysis of the 5 mm-thick coral slabs were as follows: 115 kV, 300 µA, 34.5 µm focal spot size, microfocus focal spot mode, at 12.5 fps framerate. SST records were extracted from the OISST and ERSST databases at the location of the Baler coral cores (15.7587°N, 121.6300°E).
Data source location	Institution: Philippine Nuclear Research Institute (DOST-PNRI) City/Town/Region: Quezon City, Metro Manila Country: Philippines Latitude and longitude for collected samples: 15.7587°N, 121.6300°E (Baler coral cores)
Data accessibility	With the article
Related research article	A.T. Bautista VII, S.J.M. Limlingan, M.M.T. Bauyon, A.M. Jagonoy, J.M.D. Racho, J.D.G. Valdez, B.J.T. Salon, A.J.E. Tabuso, J.K.C. Valerio, E.E. Dumalagan, H. Kusuno, F.P. Siringan, H. Matsuzaki, A historical record of the impact of nuclear activities based on 129I in coral cores in Baler, Philippines: An update, J. Environ. Radioact. 227 (2021) 106508. https://doi.org/10.1016/j.jenvrad.2020.106508

## Value of the Data

• The gray value datasets of coral cores Baler 2 and 3 provide historical records of the corals’ response to changes in the marine environment through the years and may be used in studies related to such.
• The current database relates coral 3DXCT gray values to SST. These databases provide the basis for the age models of coral cores Baler 2 and 3.
• The age model datasets of Baler 2 and 3, in turn, serve as the basis of interpretation for all current and future studies on these coral cores.
• While the current databases only relate 3DXCT gray values to SST, future investigations may look at other environmental or biological factors that affect the variability of the gray values across the coral cores’ length.
• Understanding how corals responded to changing environments in the past helps us better understand the impacts of natural- and human-driven environmental and climate changes on corals and the ecosystems they are in.

## 1 Data Description

The datasets in this article contain raw data of the coral age model, gray values, and SST matching of the Baler 2 and 3 coral cores.

There is a total of five (5) datasets:

1. B2S1O – segment 1 (top) of the coral core Baler 2 matched with OISST
2. B2S2O – segment 2 of the coral core Baler 2 matched with OISST
3. B2S2E – segment 2 of the coral core Baler 2 matched with ERSST
4. B2S3E – segment 3 of the coral core Baler 2 matched with ERSST
5. B2S3O – segment 1 (top) of the coral core Baler 3 matched with OISST

**Fig. 1:**
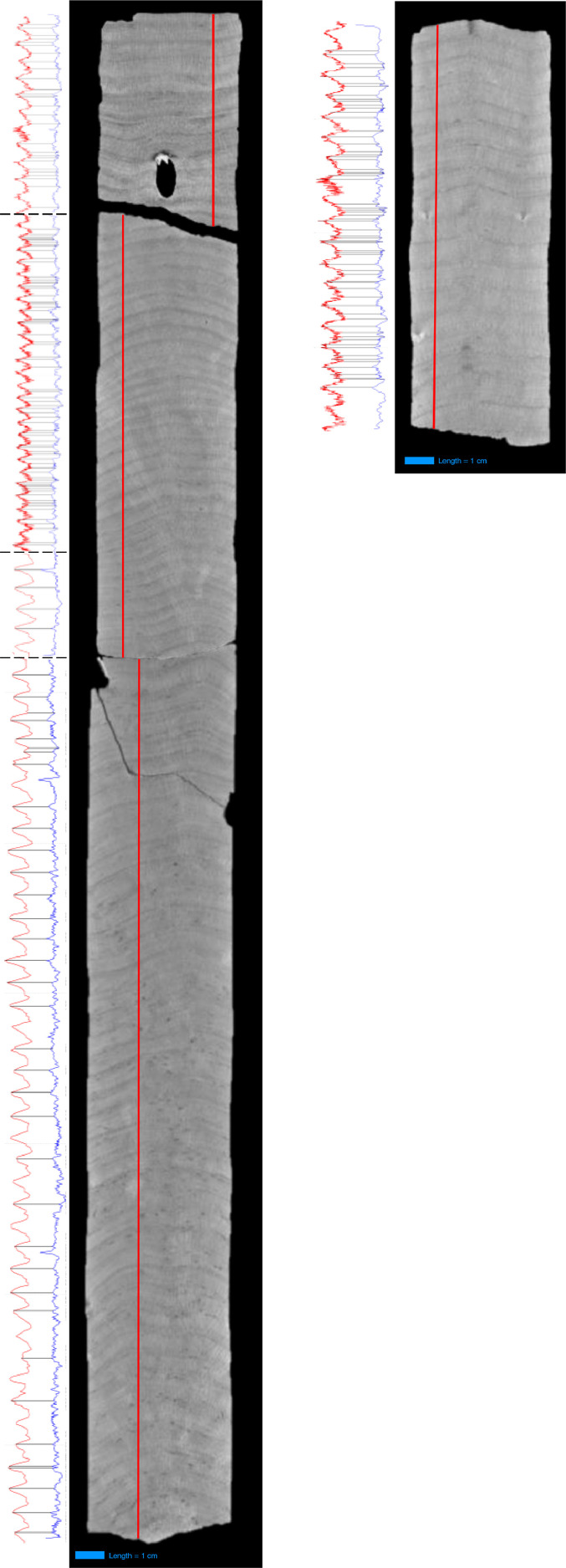
Baler 2 (longer) and Baler 3 (shorter) coral cores. Sea surface temperature variations in Baler (red curve) based on OISST [1,2] or ERSST [3], matched with coral gray values (blue curve) for age modeling using QAnalySeries [5]; Coral gray values were taken along the red line superimposed on the 2D image slice of the corals taken at 2.5 mm of the thickness axis of the slab using 3D X-ray Computed Tomography or 3DXCT; Length scale of 1 cm are shown below in blue. Image modified from Bautista et al., 2021 [4].
b. target – has 2 columns, date (in number format = number of days from January 1, 1900) and SST in degC, based on either OISST [1,2] or ERSST [3]. These are daily and monthly records of SST of the Baler study site.
c. time model – has 2 columns, pixel no. and date (in number format). All tie points between gray values and SST are recorded in this file.
d. data with ages – has 3 columns, pixel no., gray value, and date (in number format). This is the output of gray value vs. SST matching, i.e., the resulting age model of each coral core segments.

Each dataset contains four (4) .csv files:

a. data – has 2 columns, pixel no. and gray value. This is the output of the 3DXCT analysis. Gray values were taken from top to bottom of each segment, as shown in Fig. 1.

The data, target, and time model files can be directly used as input files in the QAnalySeries software [5] to view or edit the gray value vs. SST matching.

The raw X-radiographs of the Baler 2 and Baler 3 coral cores are available in the supplementary material. A composite figure of the x-radiographs alongside the 3DXCT gray values vs. SST matching is shown in Fig. 1.

## 2 Experimental Design, Materials and Methods

**Fig. 2:**
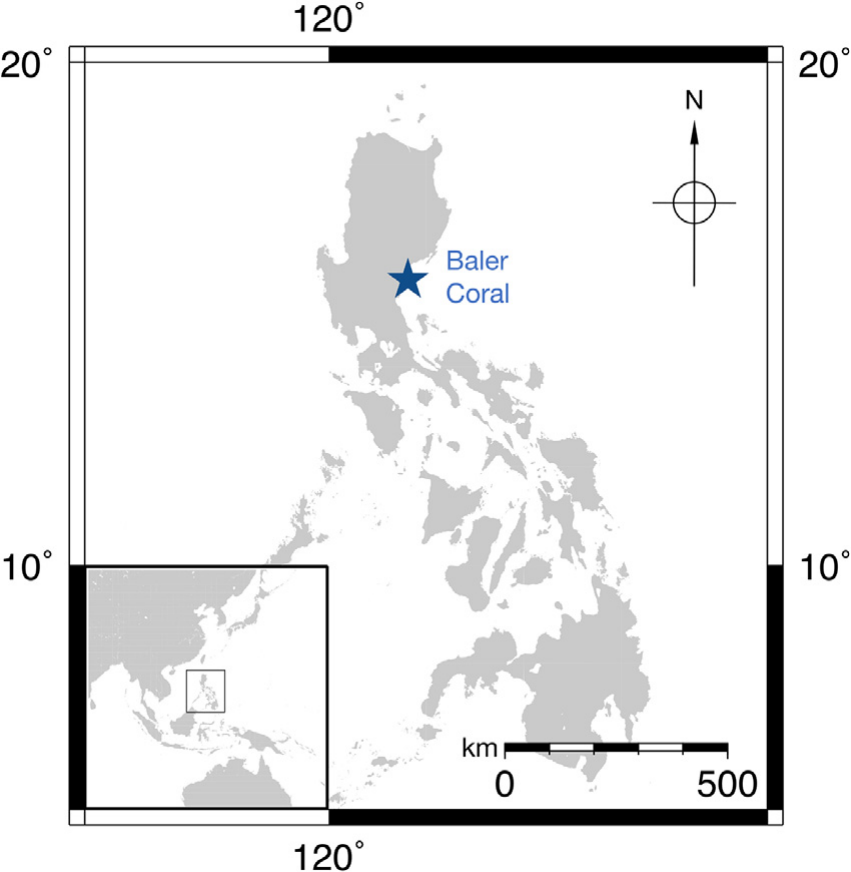
Baler Coral Site.

In October 2018, two *Porites* spp. coral cores, Baler 2 and Baler 3, were drilled from two separate coral heads (i.e., 5 m apart and both at 5 m depth) in Baler, Aurora, Philippines (15.7587°N, 121.6300°E; Fig. 2). Corals heads were selected with the following criteria: at least 1 m in radius and with the least overlapping, damage, or bleaching features among those present in the study area. Coral drilling was done perpendicular to the coral growth surface using a diver-operated hydraulic drill system with an inner diameter of 5.8 cm. The Baler 2 and Baler 3 coral cores were 50 cm and 15 cm long, respectively (Fig. 1).

The Baler 2 and Baler 3 coral cores were cut into slabs of 5 mm thickness using a circular saw cutter. These coral slabs were analyzed using 3D X-ray Computed Tomography or 3DXCT (North Star Imaging X5000) in the Industrial Technology Development Institute – Advanced Device and Materials Testing Laboratory (DOST-ITDI ADMATEL). The settings for the 3DXCT analysis of the 5 mm-thick coral slabs were as follows: 115 kV, 300 µA, 34.5 µm focal spot size, microfocus focal spot mode, at 12.5 fps framerate.

X-radiographs of all coral slabs were cross-checked against each other for consistency, and the slab with the most detail (i.e., usually the slab at the middle of the coral core) was selected for subsequent analysis. From the 3D image of the selected coral slab, a 2D image slice was taken at 2.5 mm of the thickness axis of the slab (i.e., at half-thickness), and here, gray values were determined per pixel from top to bottom of the slab. Each pixel corresponds to a spatial resolution of 54 µm. The gray value profile (i.e., peaks and troughs) across the length of the slabs were then matched with those from the SST records of the Baler site. Daily SST records from October 2018 to February 1982 were extracted from the Optimum Interpolation Sea Surface Temperature or OISST, a 1/4° daily SST record constructed from global observations via satellites, ships, buoys, and Argo floats [1,2]. On the other hand, monthly SST records from February 1982 to May 1945 were taken from the Extended Reconstructed Sea Surface Temperature or ERSST, a 2° monthly SST record based on Argo floats and sea ice data [3]. Matching of the gray values vs. SST, particularly their peaks and troughs, was done through the QAnalySeries software [5]. QAnalySeries generated the age model for the Baler 2 and Baler 3 slabs after the matching step, based on the age dates of the SST records.

## Ethics Statement

This work does not involve the use of human subjects.

This work includes the use of *Porites* spp. corals, and the study has followed the ARRIVE guidelines and is not covered by the U.K. Animals (Scientific Procedures) Act, 1986 and associated guidelines, EU Directive 2010/63/EU for animal experiments. Coral sampling followed all local protocols and regulations, as attested by Gratuitous Permit No. 0146-18 issued by the Department of Agriculture, through the Bureau of Fisheries and Aquatic Resources of the Republic of the Philippines.

## Declaration of Competing Interest

The authors declare that they have no known competing financial interests or personal relationships which have, or could be perceived to have, influenced the work reported in this article.

## Appendix Supplementary materials

Image, application 1

Image, application 2
