# Supplementary figures and images for: 3D X-ray computed tomography gray value and age model datasets of coral cores Baler 2 and 3 (Philippines)
Source: Data Brief. 2021 Jan 14;34:106755. doi: 10.1016/j.dib.2021.106755 (PMC7820376; doi:10.1016/j.dib.2021.106755)

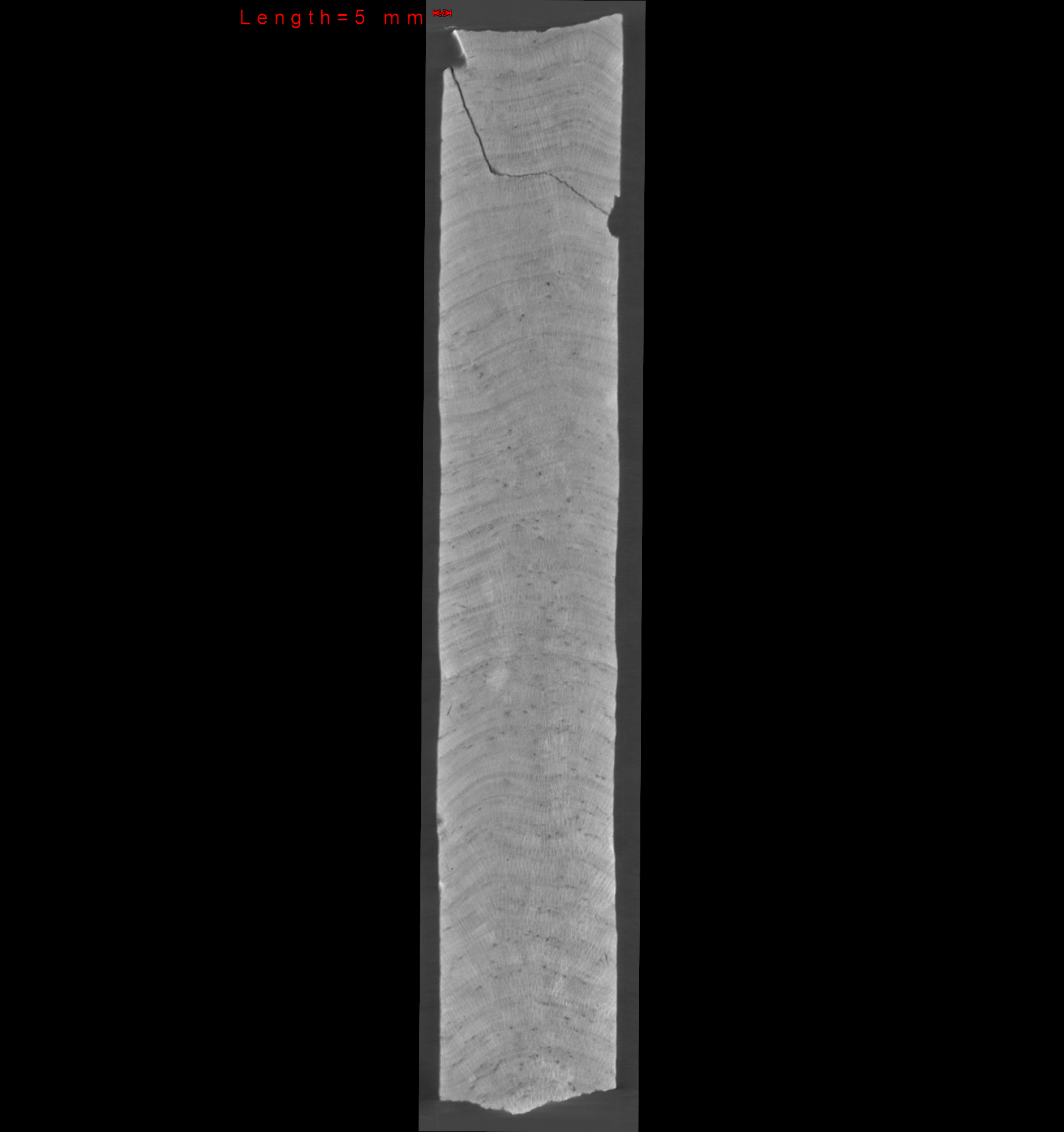

Supplement: Supplementary file 2 [file mmc2.zip › Baler Coral Images/Baler 2_3 of 3.tiff]

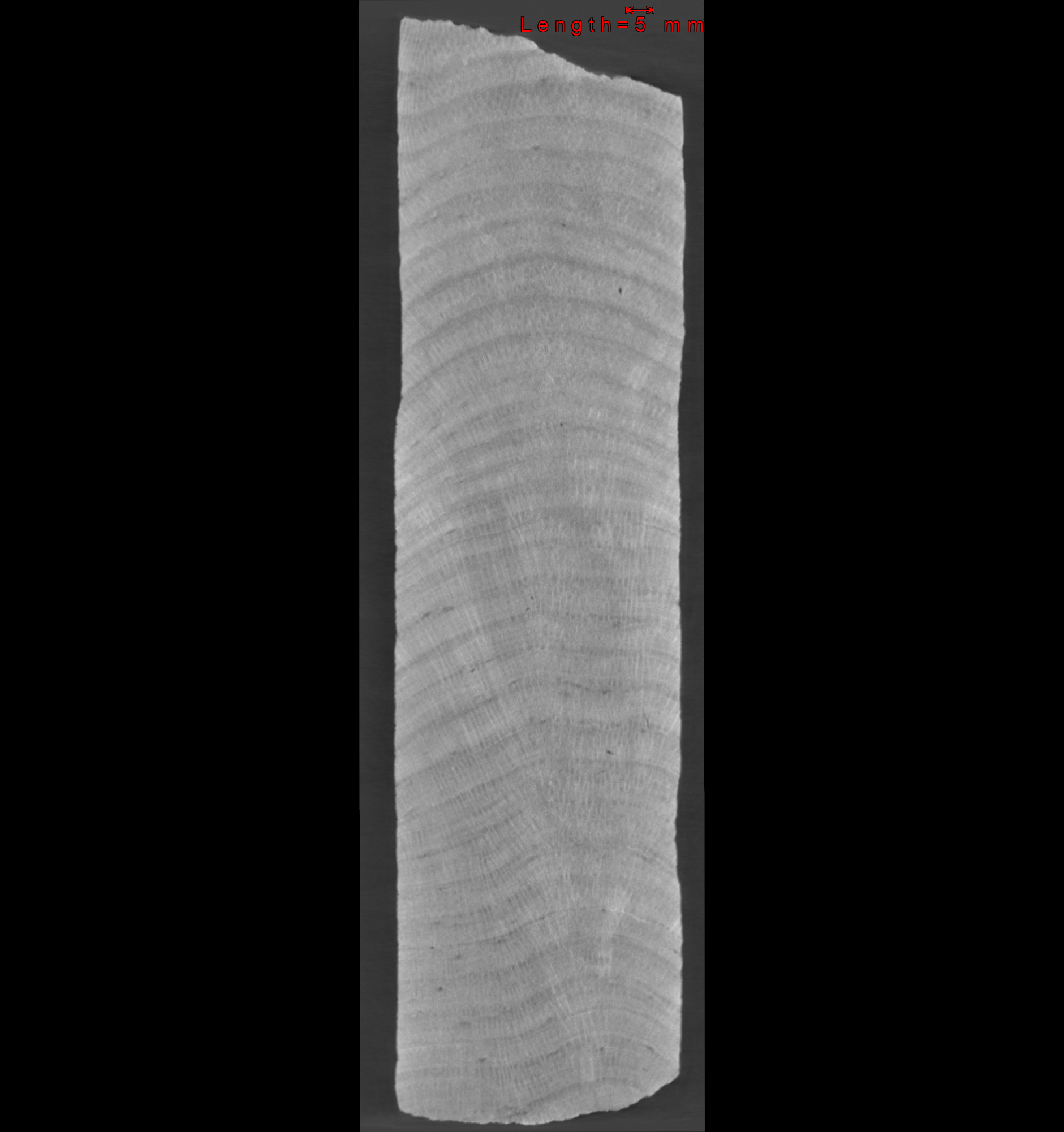

Supplement: Supplementary file 2 [file mmc2.zip › Baler Coral Images/Baler 2_2 of 3.tiff]

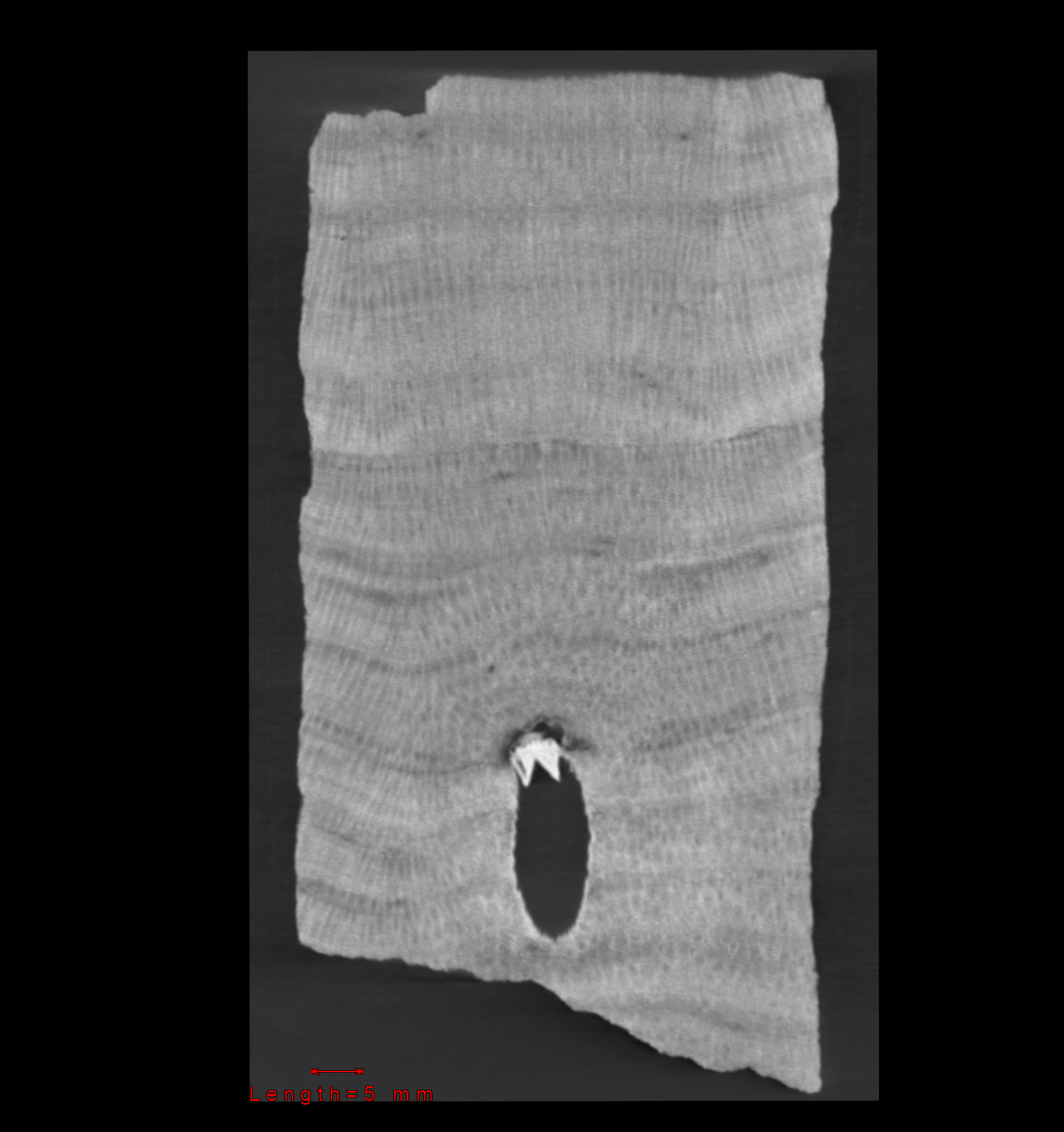

Supplement: Supplementary file 2 [file mmc2.zip › Baler Coral Images/Baler 2_1 of 3.tiff]

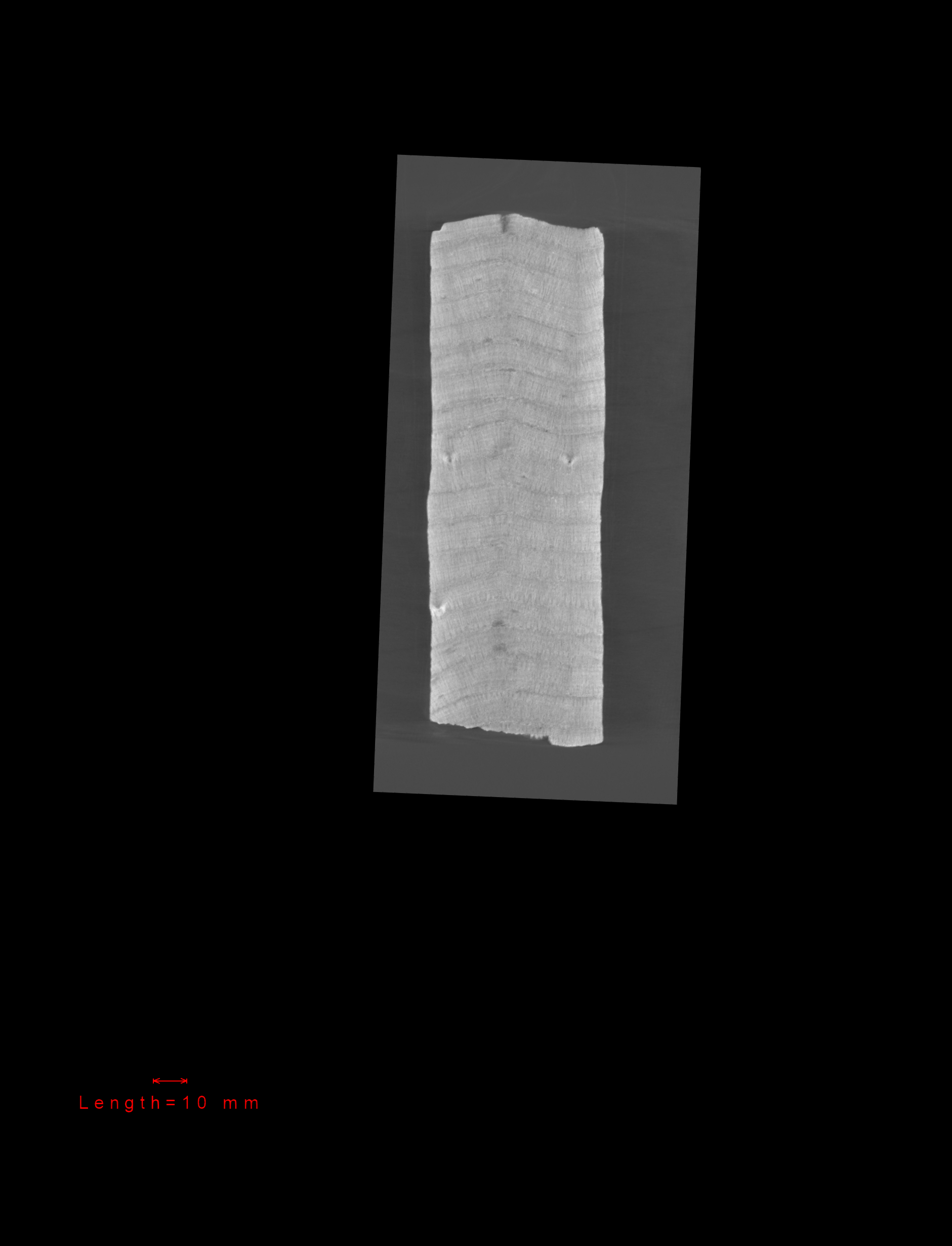

Supplement: Supplementary file 2 [file mmc2.zip › Baler Coral Images/Baler 3.tif]
